# The treasured giants: a current overview on agricultural, nutritional, bioactive, and economic potential of *Macrocybe* Species (Agaricales, Basidiomycota)

**DOI:** 10.3389/fcimb.2024.1493532

**Published:** 2024-11-18

**Authors:** Thivanka M. Peiris, Menasha Perera, Helani H. Munasinghe, Kasun M. Thambugala, Buddhika P. Dharmasena, Piyawan Suttiprapan, Ratchadawan Cheewangkoon

**Affiliations:** ^1^ Centre for Plant Materials and Herbal Products Research, University of Sri Jayewardenepura, Nugegoda, Sri Lanka; ^2^ Faculty of Humanities and Sciences, Sri Lanka Institute of Information Technology, Malabe, Sri Lanka; ^3^ Department of Botany, Faculty of Applied Sciences, University of Sri Jayewardenepura, Nugegoda, Sri Lanka; ^4^ Genetics and Molecular Biology Unit, Faculty of Applied Sciences, University of Sri Jayewardenepura, Nugegoda, Sri Lanka; ^5^ Department of Biosystems Technology, Faculty of Technology, Sabaragamuwa University of Sri Lanka, Belihuloya, Sri Lanka; ^6^ Department of Entomology and Plant Pathology, Faculty of Agriculture, Chiang Mai University, Chiang Mai, Thailand; ^7^ Agrobiodiversity in Highland and Sustainable Utilization Research Group, Chiang Mai University, Chiang Mai, Thailand

**Keywords:** commercial cultivation, diversity, edible mushrooms, nutrient composition, secondary metabolites, *Macrocybe* species distribution

## Abstract

*Macrocybe* is a well-studied genus in the family Callistosporiaceae (Basidiomycota). Currently, the genus comprises eight species with worldwide distribution. All species in this genus are relatively large compared to other edible mushrooms and are commonly consumed by locals. Cultivation methodologies have been developed for several species of the genus, including *M. gigantea*, *M. crassa*, *M. titans*, and *M. lobayensis*. These mushrooms can be cultivated in lignocellulosic wastes such as sawdust, straw, and other agro-industrial wastes. The nutritional compositions have been identified for *M. gigantea*, *M. crassa*, and *M. lobayensis*, revealing that they are rich in fibers, proteins, and various vitamins. Although these mushrooms are of culinary significance, precautions should be taken when consuming them due to their potential cyanic toxicity. In addition to being rich in different nutrients, *Macrocybe* species possess medicinal properties such as antimicrobial, antioxidant, immunomodulatory, anticancer, anti-inflammatory, hepatoprotective, and several other beneficial effects. Several species are commercially available in countries like China and Thailand, and the commercial potential is high due to the large size, taste, and long shelf life of these mushrooms. There is significant potential for cultivating species of this genus and introducing their artificial cultivation practices to various counties worldwide. Diverse value-added products can also be produced using *Macrocybe* species.

## 1 Introduction

**Figure 1:**
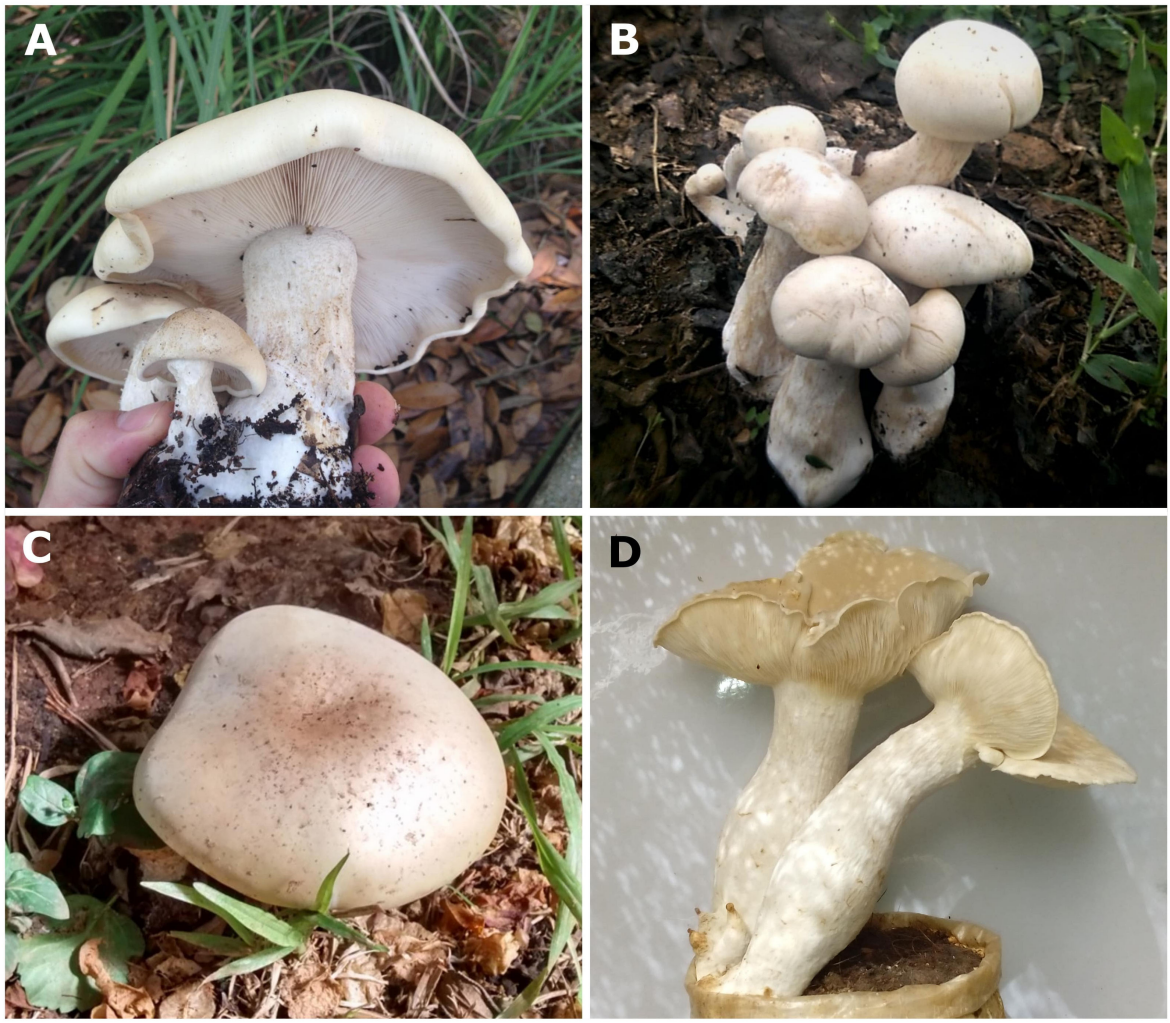
**(A)** *Macrocybe titans* (Capture credit - Logan Wiedenfeld), **(B)** *M*. *crassa* (Capture credit - Vihane), **(C)** *M*. *praegrandis* (Capture credit - Thomas TS), **(D)** *M*. *gigantea*.

The genus *Macrocybe* was established by Pegler & Lodge in 1998 under the family Tricholomataceae to accommodate nine *Tricholoma* and three *Agaricus* species *viz. Tricholoma crassum* (Berk.) Sacc., *T. giganteum* Massee, *T. lobayense* Heim, *T. pachymeres* (Berk. & Broome) Sacc., *T. praegrande* (Berk.) Sacc., *T. spectabilis* Peerally & Sutra, *T. titans* H.E. Bigelow & Kimbr. in, *T. cystidiosum* Cifuentes & Guzmán, *T. cifuentesii* Courtec., *Agaricus crassus* Berk., *A. pachymeres* Berk. & Broome and *A. praegrandis* Berk (**Figure 1**). Later Vizzini et al. (2020) introduced a novel species, *M. sardoa*, from Italy and accommodated the genus in Callistosporiaceae. Subsequently, Wijayawardene et al. (2022) placed it under Biannulariaceae Jülich. Almost all the species of this genus are bigger than most of the other edible mushroom species, and the pileus of some species extend up to 100 cm in diameter (Pegler et al., 1998). Moreover, *Macrocybe* species produce mushrooms that are considered edible and have a high agricultural potential (Teaumroong et al., 2002; Inyod et al., 2016; Verma et al., 2017; Galappaththi et al., 2022).

According to the Mushroom global market report - 2024 (https://www.researchandmarkets.com/reports/5939329/mushroom-global-market-reportproducttoc), the global mushroom industry is expected to reach a market value of USD 66.53 billion. The introduction of novel species and strains, innovative cultivation methods, and the expansion of the cultivation range are required to enhance the mushroom industry (Gamage and Ohga, 2018; Dong et al., 2022; Sangeeta et al., 2024). The main objectives of this review are to discuss the key data on various *Macrocybe* species, to explore their economic, culinary, nutritional, and medicinal significance and to critically identify and discuss the suitability of *Macrocybe* species as potential commercial crops. By analyzing the cultivation practices of the mushrooms of this genus, we aim to provide a comprehensive overview of conditions and methodologies developed in various studies and practiced in different countries. Different optimum conditions for cultivating various species of the *Macrocybe* genus, such as substrates used, environmental parameters like temperature and humidity, and cultivation practices will be discussed. Understanding these factors is crucial for enhancing productivity and ensuring the commercial viability of *Macrocybe* mushroom farming. Mushrooms of *Macrocybe* sp. have high economic potential due to their large size, quick growth, and simplicity of cultivation. The economic potential of these species is also discussed in this review. These mushroom species are highly valued in the culinary aspect due to their unique texture, flavor and versatility, and this review addresses the various culinary practices around the world and, highlights the culinary applications of *Macrocybe* mushrooms. The nutrition composition of *Macrocybe* mushrooms is another area of interest covered in this review. These mushrooms are rich in various nutrients, including proteins, vitamins, minerals, and dietary fiber, with low-calorie count and fat content. Beyond their nutritional value, *Macrocybe* mushrooms have various bioactive properties. This review also discusses the research on the medicinal potential of *Macrocybe* mushrooms, including their antioxidant, anti-inflammatory, and antimicrobial activities.

## 2 Species and their distribution

*Macrocybe* genus has been reported with eight subtropical and tropical species. Among these species, *Macrocybe titans* (H.E. Bigelow & Kimbr.) Pegler, Lodge & Nakasone, *M. lobayensis* (R. Heim) Pegler & Lodge, *M. crassa* (Sacc.) Pegler & Lodge and *M. gigantea* (Massee) Pegler & Lodge are widely distributed in various regions of the world. *Macrocybe sardoa* Vizzini, Consiglio, M. Marchetti, *M. pachymeres* (Berk. & Broome) Pegler & Lodge, *M. praegrandis* (Berk.) Pegler & Lodge and *M. spectabilis* (Peerally & Sutra) Pegler & Lodge are also included in the genus, but they have limited occurrences and documentation (**Table 1**).

**Table 1:** The worldwide distribution and occurrences of *Macrocybe* species.

*Macrocybe* species	Distribution
*M. titans*	Florida, United States of America (Bigelow and Kimbrough, 1980); Southeastern United States, Caribbean, Mexico (Cifuentes and Guzmán, 1981; Singer, 1990; Lopez and Garcia, 2018); Costa Rica (Calonge et al., 2007; Corrales and López, 2017); Parts of South America (Pegler et al., 1998); Brazil (Battistin et al, 2015); Colombia (Chivatá Bedoya, 2018; Luna-Fontalvo et al., 2023); Panama (Piepenbring, 2008); Argentina (Ramirez et al., 2017); Martinique, Trinidad, Puerto Rico, Venezuela and Ecuador (Pegler et al., 1998); India (Vrinda et al., 1997; Vrinda and Pradeep, 2006); Taiwan (Chen and Chen, 1999)
*M crassa*	India (Verma et al., 2017); Thailand (Inyod et al., 2017); Sri Lanka, Malayasia, Japan (Verma et al., 2017; Vizzini et al., 2020)
*M. gigantea*	India (Manimohan et al., 2007; Bhupathi and Subbaiah, 2019); Pakistan (Razaq et al., 2016); Nepal, China (Pegler et al., 1998), Sri Lanka (Galappaththi et al., 2022)
*M. lobayensis*	Central African Republic (Pegler et al., 1998); India, West Africa (Chakravarty and Sarkar, 1982; Vrinda and Pradeep, 2006; Verma et al., 2017); Ivory Coast, Ghana, South Africa, Benin, Democratic Republic of Congo and Nigeria (Kesel et al., 2002; Ndong et al., 2011; Härkönen et al., 2015; Bastos et al., 2023)
*M. sardoa*	Italy (Vizzini et al., 2020); India (Kantharaja and Krishnappa, 2021)
*M. praegrandis*	Brazil (Pegler et al., 1998)
*M. spectabilis*	Mauritius, Japan, and Hawaii (Pegler et al., 1998; Buyck, 2008; Hemmes et al., 2018)
*M. pachymeres*	Asia (Vizzini et al., 2020)

*Macrocybe* species commonly occur on rainforest floors, where they receive continuous rain, with an average relative humidity of around 70%, and temperatures ranging between 25–28°C (Roy and Krishnappa, 2018). *Macrocybe titans*, one of the largest known gilled mushrooms, the type species of the genus, is of tropical and subtropical distribution in the western hemisphere (neotropics). It was originally described from Florida, United States of America (Bigelow and Kimbrough, 1980). Other than the native locations, *M. titans* has been recorded in India and Taiwan. However, Vizzini et al. (2020) suggest that the specimens found in India and Taiwan are actually *M. crassa*, based on both their morphological characteristics and the known geographical distributions of the two species. In contrast to *M. titans*, other species show a Paleotropical distribution. The species *M. crassa, M. gigantea*, and *M. pachymeres* are identified as strictly of Asiatic distribution based on the study by Vizzini et al. (2020). According to their study, they hypothesize the presence of a single clade, *M. crassa* including several specimens previously identified as *M. crassa, M. gigantea* and *Lyophyllum praslinense*. This hypothesis was based on phylogenetic analysis and the close genetic relationship among these taxa. Furthermore, due to the presence of hymenial pseudocystidia which can be overlooked due to their deep subhymenial origin in various specimens. Based on the phylogenetic analysis of new collections of *Macrocybe* species together with sequences available in GenBank, Vizzini et al. (2020) have identified that those three species form a single clade and hence, have identified a single species, *M. crassa*, the older name.

**Figure 2:**
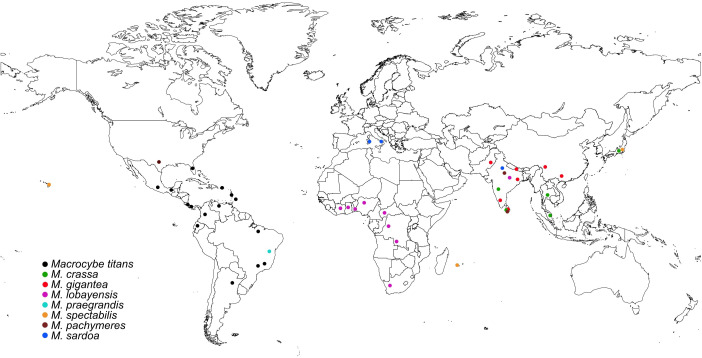
Worldwide geographical distribution of the *Macrocybe* species (Map source - https://www.mapchart.net/).

The most commonly studied *Macrocybe* species, *M. gigantea*, is distributed in tropical regions of high temperature and humidity, as well as subtropical Asian countries such as China. *Macrocybe lobayensis* distribution is mainly in tropical forests and moist-deciduous to semi-forests. They occur as scattered large caespitose clusters on humus-rich soil (Verma et al., 2017) and in open grounds, forests, savannas, or plantations (Edible fungi of tropical Africa, n.d) *Macrocybe praegrandis* is among the largest and sturdiest mushroom species, typically found growing individually or in clusters of two or more mushrooms, frequently amidst grassy areas (Pegler et al., 1998). *Macrocybe spectabilis* is always associated with sugarcane and occurs at the base of the sugarcane plants as large clusters of more than 50 basidiomata (Pegler et al., 1998). The geographical distributions of *Macrocybe* species are mentioned in **Figure 2**.

## 3 Morphological characteristics

*Macrocybe titan*s has the largest basidiocarps in the genus and can be recognized among one of the world’s largest mushrooms. Some of the largest recorded specimens of *Macrocybe* weigh about 20 kg (ABC News, 2017) and sometimes the pileus size extends to a diameter of about 100 cm and stipe can reach up to 30–40 cm in height (Karlsen-Ayala and Smith, 2020) while some recorded specimens are not that much bigger (Piepenbring, 2008; Corrales and López, 2017). According to Pegler et al. (1998), when the cap reaches maturity, its surface color changes from light ochre and many shades of buff, to finally becoming white at maturity. The spores of the mushroom are subglobose to ovoid with an apiculi (Vizzini et al., 2020). These mushrooms are often growing in clusters (Pegler et al., 1998). A comparison of major morphological characteristics often applied to distinguish these mushrooms is presented in **Table 2**.

**Table 2:** Comparison of some common characteristics of the mushrooms in the genus *Macrocybe* (Pegler et al., 1998; Vrinda and Pradeep, 2006; Bhale et al., 2019; Vizzini et al., 2020; Kantharaja and Krishnappa, 2021; Galappaththi et al., 2022).

*Macrocybe* species	Pileus surface	Pileus diameter (cm)	Context	Stipe	Taste	Odor
***M. crassa***	color varies from pale cream, yellowish brown to greyish brown and center is darker	6–24	white colored, about 3.5 cm thick at the center	15–25 ×1.4–5 cm, cylindrical, swollen at base	slightly bitter	ammoniacal
***M. gigantea***	initially white in color, and become grey with a glaucous tint, paler towards the margin	4–35	up to 3 cm thick at the center and is white, with a firm texture	15–18 × 6 cm, cylindrical and often elongated, solid, surface with same color of pileus, fibrillose-striate	–	when crushed or cut emits brewer’s grains odor
***M. lobayensis***	range from pure white to ivory white with occasional spots of ochre or reddish ochre	6–21	about 2 cm in thickness and has a consistent pure white color	stipe 5–15(–30) × 1.5–3(–6) cm, ochre-colored, often changing to a pale grey shade, and has fine wrinkles	like flour or starch, (farinaceous)	coumarin or bitter almond
***M. titans***	in maturity, its surface color changes from light ochre and many shades of buff, to finally becoming white	8–50(–100)	4 cm thick at disk, white, firm-fleshy	30–40 cm in height, cylindrical to obclavate, up to 12 cm in diameter at base, solid, surface off-white to pale grey, squamules present, often caespitose	–	strong, pleasant to disagreeable, fragrant or mushroom like
***M. pachymeres***	hemispherical, convex, surface pale ochracous to greyish brown, paler towards the margin	4–10	2.5 cm thick, white	5–11 × 1.5–4 cm, cylindrical to obclavate, with swollen base, solid, surface pale grey, covered with small squamulose	–	–
***M. spectabilis***	convex, umbonate to applanate, surface pure white to ‘Buff’, glabrous, smooth, silky, dry	7–40(60)	1–2.5 cm, pristine white in color with a firm texture thick, abruptly thinner at the margin	5–30 (–40) × 4–7 cm cylindrical to obclavate, solid becoming finally fistulose, surface pure white	–	strong, cyanic
***M. praegrandis***	convex, finally depressed at center, surface cream colored, smooth, with an irregular, wavy margin	20–50	1–2 cm thick and is pure white and firm in color	17–30 × 2.5–4 cm, solid, surface cream colored, smooth, with a basal cottony mycelium	sweetish to mild unpleasant taste, stipe -slightly bitter taste	pleasant order
***M. sardoa***	convex, flat-convex, incurved at first and then decurved, sinuous, lobed, surface first pure white but turning cream-ochre with time	15–25(–35)	15–25(–40) cm	10–15(–20) × 4–6(–8) cm Same color as pileus, compact and solid, usually cylindrical	mild	pleasant and sweetish when young, unpleasant and nauseating (compost-like) when mature

When considering *M. crassa*, the basidiocarps can be weighted up to 1.25 kg (Pegler et al., 1998). *Macrocybe gigantea* together with *M. crassa* are considered as the two largest tricholomatoid agarics in South Asian region (Pegler et al., 1998; Vizzini et al., 2020) and *M. gigantea* is distinguished from *M. crassa* from the hymenophore color straw yellow which is white in *M. crassa* (Razaq et al., 2016; Vizzini et al., 2020). *Macrocybe pachymeres* which only have two specimens observed from Sri Lanka and India, is closely resembles the morphology of *M. titans* but differs only for the uncertain presence of pseudocystidia and remained as a separate taxon until new type materials are observed (Pegler et al., 1998; Vizzini et al., 2020).

## 4 History of consumption and cultivation

The recognition of the edibility of the mushrooms of the genus *Macrocybe* extends back to several centuries, although historical records on their consumption and cultivation are rare. Amidst the recent cultivation records, these mushrooms can be considered only wild-picked and not cultivated. Among these species, *M. titans* is recognized as a prominent edible mushroom (Kimbrough, 2000; Bessette et al., 2007; DeLong and Brewer, 2019) and is traditionally used in Colombia (Vargas et al., 2022). Historically, the cultivation of this mushroom has not been practiced, and successful artificial cultivation methods were introduced by Stijve (2004) and Duong et al. (2017). *Macrocybe gigantea*, *M. spectabilis* and *M. lobayensis* have also been utilized as wild picked edible mushrooms (Boa, 2004). In China, fresh *M. gigantea* mushroom collected from the wild is eaten boiled, mainly prepared as soups and rarely consumed raw (Falandysz et al., 2020).

According to the classification by Li et al. (2021), *M. crassa, M. gigantea, M. lobayensis*, *M. titans* and *M. praegrandis* are classified as ‘edible with confirmed edibility’ and *M. spectabilis* as ‘edible with confirmed edibility but with conditions.’ *Macrocybe spectabilis* should not be consumed raw due to the presence of cyanic compounds and is excellent when cooked (Rammeloo and Walleyn, 1993). According to Dutta and Acharya (2014), *M. crassa, M. gigantea, and M. lobayensis* (identified as ‘dhoodh chhatu’ or ‘boro dhoodh chhatu’ in Indian local language) are consumed by local and indigenous communities of the Lateritic region and the coastal region of the West Bengal of India. They prefer the mushrooms cooked with mustard oil and spices to make them more flavorful. Also, these three species are available in local markets of these regions either in fresh or dried form. *Macrocybe crassa* is also consumed as wild picked mushroom by the Santal people of India and sold in roadside markets (Pradhan et al., 2010). In Sri Lanka, *M. gigantea*, known locally as ‘pol hathu’ in Sinhalese, is consumed as a delicacy and primarily occurs in the country’s wet zone. Locals use an *in-situ* cultivation strategy by placing the discarded mushroom pieces into decaying matter (leaf litter), with the aim of encouraging the mushroom to reappear, although this method has a low success rate. *Macrocybe lobayensis* is frequently found in Africa (Rammeloo and Walleyn, 1993), and this mushroom is available in local markets as fresh or dried (Zoberi, 1972). In Ghana, *M. lobayensis*, known locally as ‘inku adjinaku,’ is highly preferred and consumed, especially in the northern regions of the country (Kesel et al., 2002). This mushroom is also consumed in Togo (Kamou et al., 2015) and is popular among various ethnic groups in Burkina Faso (Guissou et al., 2008). In Nigeria, *M. lobayensis* is considered a delicacy and is sold in local markets, where it is typically cleaned and peeled before being offered for sale (Zoberi, 1979). In Thailand, *M. crassa* is a common edible mushroom that is identified by different local names such as ‘hed-tin-rad’, ‘hed-jan’, ‘hed-hua-sum’ or ‘hed yai’ (Petcharat, 1996). This mushroom is rare and appears annually during the rainy season, creating a high demand for it (Teaumroong et al., 2002). This mushroom is consumed in Laos as soups and barbecues (Souvannakhoummane, 2021).

## 5 Current cultivation practices

**Figure 3:**
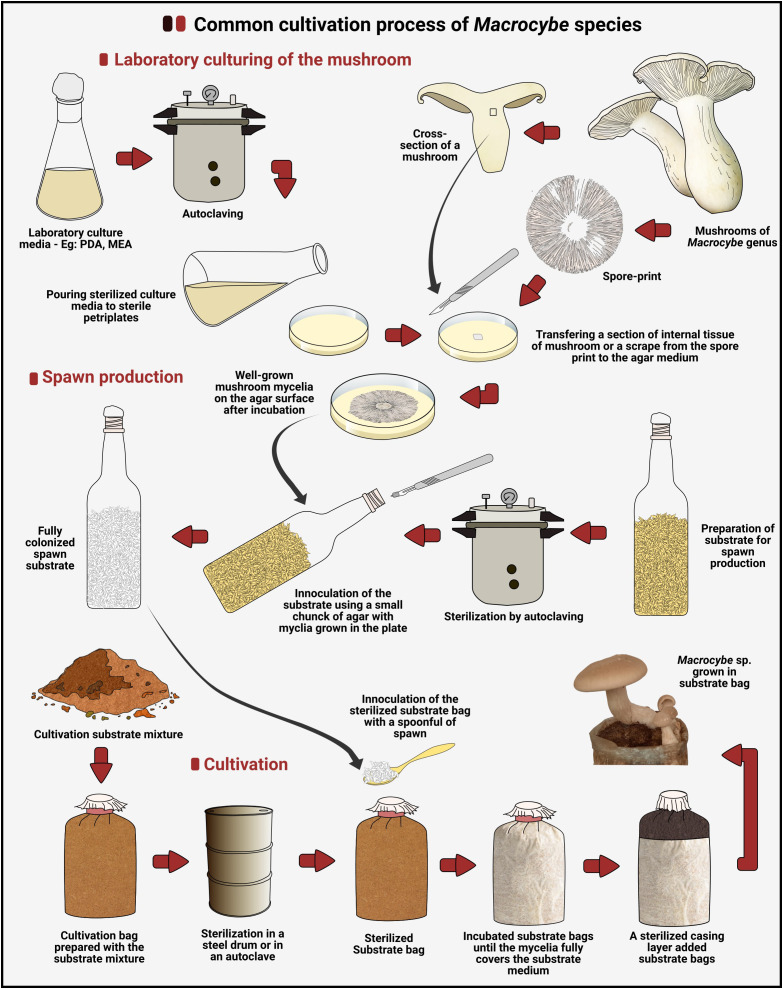
The commonly applied pipeline for cultivation of *Macrocybe* spp. (Designed using Inkscape v1.1).

*Macrocybe* species are primarily cultivated in various countries, including China and Thailand through domestic cultivations, with industrial cultivation being rarely documented. The limited cultivation and novelty of cultivation methods may contribute to the scarcity of documentation. Despite being grown on a small scale domestically, there is a notable demand for mushrooms of this genus. All the mushroom species in this genus can be successfully cultivated in lignocellulosic wastes such as saw-dust and straw in bag cultivation method. Among the species of the genus, *M. gigantea* is the most widely cultivated, although its cultivation is not popularized and in a much smaller scale compared to other commercial mushrooms. Anyway, the conditions for growing this species have been optimized (Galappaththi et al., 2022). The cultivation technology for mushrooms in this genus follows similar principles to those used for other mushroom species. The cultivation process begins with culturing the mushroom in laboratory media. Subsequently, spawns are prepared from this culture. The actual cultivation is then carried out by using the prepared spawn as the inoculum, which is introduced to a lignocellulosic substrate (**Figure 3**).

In laboratory culturing, pure cultures of the mushroom should be prepared. Here, the mushrooms are cultured on a diverse range of culture media such as Malt Extract Agar (MEA) and Potato Dextrose Agar (PDA). In culturing of *Macrocybe* species, Galappaththi et al. (2022) have successfully utilized PDA for *M. gigantea*. Chen and Yang (2009) have shown that a PDA medium mixed with inorganic salts and yeast extract is more suitable for culturing *M. lobayense* than PDA, and a wort peptone medium was identified as the most suitable. After selecting and preparing laboratory cultures, the next step is spawn preparation. Different spawn preparation techniques are used for various mushroom species, with grain spawn technology being the most commonly applied method for *Macrocybe* spp. In this method, the mycelial discs from prepared cultures are carefully inoculated onto the previously prepared grains. Different grain types are used for this and Galappaththi et al. (2022) have used paddy seeds in spawn preparation of *M. gigantea* with promising suitability. Devi and Sumbali (2021b) have checked the suitability of three different grain types for spawn development, and their impact on the growth and yield of *M. gigantea*. Accordingly, it has been concluded that pearl millets (*Pennisetum glaucum*) are better for the spawn production of *M. gigantea* than wheat (*Triticum aestivum*) and maize (*Zea mays*). Different lignocellulosic substances are utilized in the cultivation of *Macrocybe* spp. According to Xingkui et al. (2006), rapeseed hulls and cottonseed husks are suitable as substrate media for the cultivation of *M. gigantea*. Devi and Sumbali (2021a) have tested various agro-industrial wastes for the cultivation of *M. gigantea*, including wheat (*Triticum aestivum*) straw, bajra (*Pennisetum glaucum*) stalk, paddy (*Oryza sativa*) straw, maize (*Zea mays*) cobs and stalks. They have identified that those substrates are suitable for cultivating this mushroom, with the highest biological efficiency achieved using maize stalks. Han et al. (2011) have cultivated *M. lobayensis* on *Flammulina velutipes* spent substrates and have obtained a biological efficiency of 111.79%. Megha et al. (2019) have cultivated *M. gigantea* on wheat straw. The laboratory culturing conditions, spawn preparation methods, and cultivation conditions for different *Macrocybe* spp. across various studies are detailed in **Tables 3**–**5**, respectively.

**Table 3:** Various conditions and parameters that are applied in laboratory culturing of *Macrocybe* species.

Species	Media	Temperature	pH	Light	Reference
*M. gigantea*	YEA, WGA, CA, PDA, MEA, YGA, CDA, Maize EA, DM, PEA, YPD, GPYEA, PMA, EA, GPA - Solid media RS, GAM, MPB, GPM, PW, AHM, KCM, DM, CDM, YGM, PDB, PBD, RSM, WMSM, MF, BM - Liquid media	30 ± 2°C	5.0	light and dark conditions on both solid and liquid media	Kaur et al. (2023)
	PDA, MEA, SDA, CZA	25 ± 2°C	5.6–7.5	–	Roy and Krishnappa (2018)​
	SMYA	25–35°C	–	–	Niihara (2002)
	PDA	27–30°C	–	dark conditions	Galappaththi et al. (2022)
	PDA	25 ± 2°C	–	–	Nirmala and Siva (2023)
	PDA, MEA, CDA	27 ± 2°C	5–10	blue, green, red, yellow and artificial day light	Suman et al. (2018)
	SDA	35°C	7	–	Pamitha (2014)
	MEA, PDA, GPA, SDA, CEA,	20–30°C	–	–	Ghafoor et al. (2022)
	Hennerberg medium – synthetic liquid medium GCMY (glucose, casamino acid, malt extract, and yeast extract) medium of natural liquid medium	30°C	5	–	Kinjo and Miyagi (2006)
	PDA, MEA	20–25°C	–	–	Devi and Sumbali (2021b)
*M. crassa*	PDA	20–30°C	–	–	Payapanon and Srijumpa (2008)
	PDA	30°C	–	–	Teaumroong et al. (2002)

YEA, Yeast Extract Agar; WGA, Wheat Grain Agar; CA, Coriander Agar, PDA, Potato Dextrose Agar; MEA, Malt Extract Agar; YGA, Yeast Glucose Agar; CDA, Czapek Dox Agar; Maize EA, Maize Extract Agar; DM, Dimmick Medium; PEA, Pea Extract Agar; YPD, Yeast Potato Dextrose Agar; GPYEA, Glucose Peptone Yeast Extract Agar; PMA, Potato Malt Agar; EA, Elliott Agar; GPA, Glucose Peptone Agar; RS, Richards solution; GAM, Glucose Asparagine Medium; MPB, Maltose Peptone Broth; GPM, Glucose Peptone Medium; PW, Peptone Water; AHM, Asthana and Hawker Medium; KCM, Koser Citrate Medium; DM, Dimmick Medium; CDM, Czapek Dox Medium; YGM, Yeast Glucose Medium; PDB, Potato Dextrose Broth; PBD, Pea Broth Dried; RSM, Rye Seed Medium; WMSM, Will Mineral Salts Medium; MF, Mehrlich Formula; BM, Bilai Medium; SDA, Sabourauds Dextrose Agar; CZA, Czapek Dextrose Agar; SMYA, Skim Milk Yeast extract agar; CEA, Compost Extract Agar.

**Table 4:** Different conditions and parameters that are applied in the spawn preparation of different *Macrocybe* species.

Species	Substrate	Temperature	Substrate moisture	pH	Light	Reference
*M. gigantea*	Paddy grains	27–30°C	-	-	dark conditions	Galappaththi et al. (2022)
	Sorghum grains, paddy straw, vermicompost and sand (1:1)		-	-	-	Nirmala and Siva (2023)
	sorghum, wheat, and barley grains	30°C	-	-	-	Ghafoor et al. (2022)
	sorghum grains	28 ± 2°C	-	-	-	Prakasam et al. (2011)
	bajra (*Pennisetum glaucum* L.), wheat (*Triticum aestivum* L.) and maize (*Zea mays* L.)	28 ± 2°C	-	6–7	-	Devi and Sumbali (2021b)
*M. crassa*	peanut husks and sawdust fermented by horse dung or corn waste: sawdust: blended corn (15:4:1)	28–30°C	60%	-	-	Teaumroong et al. (2002)

**Table 5:** Different conditions and parameters that are applied in cultivation of *Macrocybe* species and biological efficiencies.

Species	Substrate	Temperature	Substrate moisture	Humidity	Light	pH	Yield (weight per substrate bag/weight of substrate bag)	Biological Efficiency	Reference
***M. gigantea***	castanopsis sawdust, bark compost with wheat bran, broad-leaved sawdust (Japanese knotweed, Taiwanese alder, ginnem): rice bran: husuma = 8:0:5:0:5 (volume ratio)	25–35°C	65%	90%	–	6.3	15,438–20,043 g total yield in an open field. In air condition room - 130-181 g/bag	17.3–17.5%	Niihara (2002)
	rubber sawdust, rice bran, CaCO_3_, gypsum and MgSO_4_	27–30°C	36%	60–75%	natural light	–	1078 g/bag	43%	Galappaththi et al. (2022)
	Bajra, wheat, maize grains and paddy straw (wheat and paddy straw are used for the spore preparation)	25-35°C	60%	80–90%	light of 8-10 hours	6–7	331.3–343.6, 332.0–327.0, 320.0–334.6 (g/500g)	66.7%,66.4%,64.0%, 65.4–68.7%	Devi and Sumbali (2021b)
	agricultural wastes such as paddy straw, wheat straw, and maize cobs	–	–	–	–	–	–	55%	Nirmala and Siva (2023)
	paddy straw, bamboo leaves and neopeat.	30 – 35°C	–	–	–	7	488.50–724.00 (g/kg)	72.40%, 56.75%, 54.60%	Pamitha (2014)
	Tea waste & wheat straw, Pure wheat straw, Sawdust & wheat straw, Tea waste & sawdust, Pure sawdust and Pure tea waste	28–30°C	65%	70–85%	–	–		86.77%, 79.85 %, 70.84%, 46.9%, 40.3%, 38.86%	Ghafoor et al. (2022)
	Sawdust, wheat bran, Hannoki	–	–	–	–	–	–	–	Kinjo and Miyagi (2006)
	paddy straw, casing soil	30–35°C	–	75–80%	–	–	820.0–884.8 g/500 g	164.0–176.9%	Prakasam et al. (2011)
***M. crassa***	rubber tree sawdust, fine rice bran, MgSO_4_.7H_2_O, CaO (100:3:0:2:1), sorghum grains	Ambient room temperature 20–25°C	–	65–85%	Dark Condition	–	189.00–215.10 g/0.6 kg	34.16–59.26%	Inyod et al. (2016)
	rubber tree (*Hevea brasiliensis*) sawdust mixed with fine rice bran, magnesium sulfate (MgSO_4_.7H_2_O) and calcium oxide (CaO) in the ratio of 100:3:0.2:1	25°C	65%	85–90%	Dark condition	–	–	–	Verma et al. (2017)
	rubber sawdust, soybean residue, corn meal, fine rice bran, water hyacinth, soybean residue	26.05–28.10°C (Average substrate temp.) 30.11–34.03°C (Average air temp.)	–	–	–	6.4	–	–	Sornprasert et al. (2017)
	spent mushroom, substrate in *Pleurotus eryngii* cultivation, fine rice bran, MgSO_4_.H_2_O, CaO, and fine corn seeds 100:3:0.2:1:0.5 (w/w)	–	50%	85%	–	–	237.21 g/0.6 kg	65.89%	Inyod et al. (2023)
	mung bean husks, potting soil and mung bean husks mixed with potting soil (1:1, v/v)	–	60–70%	–	–		–	–	Teaumroong et al. (2002)
***M. titans***	shred agricultural by-products such as grasses, cereal straws, and hulls	above 25°C	–	–	–	–	–	–	Cotter and Tradd (2014)

When cultivating the species of *Macrocybe*, a casing layer needs to be applied to initiate the pinheads of the mushrooms. Niihara (2002) has used weathered pumice as the casing in the cultivation of *M. gigantea*. Loamy soil, leaf debris, and charcoal, at a ratio of 10:2:1 (w/w) has been used by Inyod et al. (2023) in the cultivation of *M. crassa*.

Different biological efficiencies have been observed when cultivating these species in different substrate media. The biological efficiency of cultivation as observed by Prakasam et al. (2011) for the cultivation of *M. gigantea* ranges from 164 to 174%. It is 86.77 ± 0.035% for the same mushroom species when cultivated in tea waste and wheat straw (Ghafoor et al., 2022). The biological efficiency for *M. crassa* was 29.11% when cultivated in a rubber saw-dust-based substrate medium.

## 6 Global industrial potential as a food product

Mushrooms are sold fresh, dried, or canned (Amin et al., 2014) and are readily perishable when fresh. Preservation extends their shelf-life (Ho et al., 2020) and canned mushrooms are popular in soups and stews (Ho et al., 2020), while dried ones are used in instant foods (Süfer et al., 2016). Powdered mushrooms also have a high market potential as a food product (Ho et al., 2020). All currently consumed *Macrocybe* species possess desirable characteristics for commercial cultivation, including large basidiocarps and extended shelf life. Commercial cultures and spawns are available for *M. crassa*, *M. titans*, and *M. gigantea*. With the increasing demand for edible mushrooms due to their nutritional content, flavor, meaty texture, and medicinal properties (Moore and Chiu, 2001), *Macrocybe* species demonstrate significant potential for both cultivation and commercialization. *Macrocybe crassa* features a large fruiting body with a meaty texture and a delicious taste. Even though it is cultivated commercially, it is not done on a large scale (Payapanon and Srijumpa, 2008). This mushroom can be commercialized as a dry powder (Inyod et al., 2016) and has the potential to be used for food fortification. In China, *M. gigantea* is famous for two different types of cuisines, soups using the mushroom’s fresh fruit bodies, and ‘quick-fire stir-fry’ using mushrooms with meat and vegetables (Wiejak et al., 2014). In India, this mushroom is well-regarded as a food source (Razaq et al., 2016) and has a significant market potential. It is also cultivated in Yunnan province of China (Mao, 2000) other than Thailand, India, and Sri Lanka. The long shelf life of mushrooms in this genus greatly enhances their market potential. Suman and Sharma (2018) have tested several storage methods to extend the shelf-life of *M. gigantea* and to increase its commercial viability. According to their findings, these mushrooms can be successfully stored in a solution of 2% salt, 2% sugar, 0.3% citric acid, 0.1% potassium metabisulphite, and 1% ascorbic acid without blanching mushrooms to increase their shelf-life. The mushrooms can be stored for about 17 days under frozen conditions. The marketing of this mushroom is heavily dependent on its storage period, making the determination of its shelf life essential (Li et al., 2015).

Mushroom species of the *Macrocybe* genus are rarely used in industrial applications beyond mushroom farming. *Macrocybe crassa* is capable of producing amylolytic enzymes, although not a prominent producer (Wisniewski et al., 2010). Strain optimization and culture conditions optimization could facilitate enzyme production. With further research on optimizing the mushroom strain and enzyme production conditions, these enzymes could be synthesized on an industrial scale using this mushroom species.

## 7 Nutrient compositions

Mushrooms are a significant food source that possesses high nutritional value and are consumed worldwide based on their palatability and nutritional benefits (Miles and Chang, 2004; Dawadi et al., 2022; Ayimbila and Keawsompong, 2023; Navarro-Simarro et al., 2024). According to much research, edible mushrooms are rich in many nutritional constituents such as dietary fibers, polysaccharides such as β-glucans, glycoproteins and peptides, vitamins, minerals, fats, and fatty acids (Assemie and Abaya, 2022; Dimopoulou et al., 2022). These mushrooms are particularly rich in proteins and dietary fibers, while carbohydrates and fats are present in relatively lower amounts (Manzi et al., 1999, Manzi et al., 2001). *Macrocybe titans* are included with sugars such as glucose, galactose, fructose, and mannose (Carbonero et al., 2006). According to Zhang et al. (2017), the ratios of total essential amino acids to total amino acids of *M. gigantea* were close to or exceeded the standards set by the FAO/WHO. Considering the *Macrocybe* genus a limited number of studies have been carried out to determine the nutritional composition. The amino acid profile, fatty acid composition, and vitamin content of these mushrooms have yet to be characterized. However, it is generally considered that *Macrocybe* mushrooms are good sources of nutrients. Different studies have been conducted to identify the nutrient composition of *Macrocybe* species (**Table 6**) and the mineral composition (**Table 7**).

**Table 6:** Nutrient composition of different *Macrocybe* species based on dry weight.

Species	*M. gigantea*							*M. crassa*			*M. lobayensis*		
**Moisture (% based on total weight)**	89.44 ± 0.16	85.3	71.13 ± 1.14		82.6 ± 0.05	90.4 ± 0.5	86.20	12.76	13.6	14.35			93.9
**Ash (%)**	9.90 ± 0.04	0.8	4.53 ± 0.32		6.23 ± 0.05		8.32	12.04	11.2	12.07	08.48 ± 0.32		9.3
**Fat (%)**	3.20 ± 0.04	1.6	1.27 ± 0.04	2.91		3.1	0.91	1.86	4.89	1.76	3.28 ± 0.15		
**Protein (%)**	37.60 ± 0.09	2.3	31.04 ± 0.76	35.28	16.4 ± 0.36 (mg/g)	16.7	32.9	12.79	14.3	30.89	20.66 ± 1.24	4.5 ± 0.2	13.9
**Total sugar (%)**				53.74	38.6 ± 0.30 (mg/g)								
**Reducing sugars (mg/g)**					8.41 ± 0.79								7.6
**Carbohydrate (%)**	32.00 ± 0.40	10	52.01 ± 0.04				11.8	62.99	65.3	74.75	57.55 ± 2.04	27.71 ± 3.66	
**Fibre (%)**	5.90 ± 0.04		10.69 ± 1.55	8.76		12.5	20.71	2.36	1.67	1.74			7.2
**Energy (kcal/100 g)**	307 ± 0.40	63.6	336.81 ± 3.55						35.2	371.76	342.36 ± 14.47		
**Amino acids (g/100 g)**				18.67									
**β– glucans (%)**								44.91	37.6	33.97 (g/100g)		11.4 ± 0.19	
**Vitamin D** **(µg/g)**	2.85 ± 0.009												
**Vitamin B1** **(mg/100 g)**				0.248									
**Vitamin B2** **(mg/100 g)**	0.38 ± 0.009			2.12									
**Niacin** **(mg/100 g)**	51.50 ± 0.090												
**Vitamin C** **(mg/100 g)**	33.00 ± 0.400												
**Reference**	Khumlianlal et al. (2022)	Galappaththi et al. (2022)	Das (2017)	Liu et al. (2007)	Gaur and Rao (2017)	Giri et al. (2013)	Prakasam et al. (2011)	Inyod et al. (2016)	Ayimbila et al. (2022)	Inyod et al. (2023)	Chibou Ousmane et al. (2024)	Ghosh et al. (2019)	Gbolagade et al. (2006)

**Table 7:** Mineral composition of different *Macrocybe* species based on dry weight.

Units	*M. gigantea*								*M. crassa*			*M. lobayensis*	
	ppm	mg/100 g	mg/kg	mg/kg	mg/100 g	mg/kg	mg/g	mg/100 g	mg/kg	mg/kg	mg/kg	mg/100 g	mg/100 g
**Ca**	2.778 ± 0.153	26.4 ± 0.10	470 ± 130	3.74 ± 0.16	0.015%		1.54	49.34	433.04	433	499.09	35.67 ± 3.51	1.3
**Cu**	5.771 ± 0.020	2.08 ± 0.07	13 ± 7	0.55 ± 0.03	18.11	1.10	0.524		13.07			2.62 ± 0.09	0.09
**Fe**	7.467 ± 0.199	17.6 ± 0.43	79 ± 23	0.27 ± 0.03	78.72	5.60		33.21	213.44	420	282.14	6.25 ± 0.65	0.30
**K**	210.380 ± 0.215		1300 ± 450		3.11%		69		35,300	35300	47800	439.67 ± 12.70	15.5
**Mg**	4.638 ± 0.064	10.7 ± 0.26	550 ± 170	21.63 ± 0.14	0.065%				651.15		1474.00	41.83 ± 2.74	0.4
**Mn**	0.225 ± 0.016	2.23 ± 0.15	5.9 ± 2.5	0.35 ± 0.001	5.13	1.18			13.0	1.09	16.16		0.30
**Na**	4.488 ± 0.055		580 ± 94						828.97	930	960.72	72.09 ± 2.45	2.2
**P**	0.016 ± 0.0005	601.5 ± 0.30			0.96%				5300	8600	9100	1298.35 ± 24.70	9.7
**Cr**		0.24 ± 0.04	0.65 ± 0.37	0.10 ± 0.008									
**Zn**	4.149 ± 0.036	13.3± 0.20	160 ± 49	0.15 ± 0.02	7.77	1.38	0.20	37.08	37.65	85.0	58.72	3.05 ± 0.14	0.80
**Co**		0.39 ± 0.08	0.29 ± 0.26										
**Se**								0.051					
**Ni**				0.09 ± 0.006									
**Cd**				0.30 ± 0.01									
**Reference**	Khumlianlal et al. (2022)	Gaur et al. (2016)	Liu et al. (2012)	Das (2017)	Roy et al. (2022)	Prakasam et al. (2011)	Ghafoor et al. (2022)	Liu et al. (2007)	Inyod et al. (2016)	Ayimbila et al. (2022)	Inyod et al. (2023)	Chibou Ousmane et al. (2024)	Gbolagade et al. (2006)

## 8 Bioactivities, nutraceutical potential, and health implications

Mushrooms have long been valued worldwide for their culinary and nutritional benefits. In addition, they have been used as medicinal sources for centuries, with recent research confirming their various medicinal properties (Sganzerla et al., 2022). As medicinal sources, mushrooms are used as dietary supplements, nutraceuticals, or myotherapy products (Wasser, 2014). Mushrooms have various medicinal properties such as antimicrobial, anti-inflammatory, immunomodulatory, antidiabetic, cytotoxic, hepatoprotective, anticancer, antioxidant, antiallergic, antihyperlipidemic, and prebiotic properties (Jeitler et al., 2020).

*Macrocybe* spp. also have of many medicinal and nutraceutical properties and are used in various medicinal applications. Among these species, *M. crassa*, is prominent in several pharmacological properties (Inyod et al., 2022). This mushroom contains high β-glucan content (Ayimbila et al., 2022) together with lipids, proteins and phenolic compounds which are considered the majority of the bioactive mycochemicals (Cateni et al., 2022). β-glucans have been demonstrated to have immunomodulatory, antioxidant, anti-inflammatory, and analgesic properties (Bobek and Galbavy, 2001). Also, these mushrooms are rich in minerals essential for metabolic reactions, sensory stimulation, rigid bone formation, and regulation of water and salt balance in the human body. They are also rich in K and P which are linked to the development of teeth and bones and are also important for healthy, smooth muscle contraction (Soetan et al., 2010). *Macrocybe crassa* shows potential antimicrobial activity against pathogenic Gram-positive and Gram-negative bacteria (Khatua and Acharya, 2014a; Inyod et al., 2022). This mushroom species is rich in pyrogallol, flavanol, benzoic acid derivatives, cinnamic acid, and its derivatives and shows antioxidant and antimicrobial activities (Khatua and Acharya, 2014b). According to Inyod et al. (2022), hot water extract of *M. crassa* can resist gastric enzyme hydrolysis and human pancreatic α-amylase enzyme in the digestion system, which is helpful to maintain the gastro digestion system and has the potential to promote lactic acid bacteria. According to Ayimbila et al. (2022), the action of *M. crassa* on gut microbiota is attributed to the short-chain fatty acids and propionic acid produced by the mushroom. This mushroom also has antioxidant potential (Khatua and Acharya, 2014a; Acharya et al., 2015; Pal et al., 2019; Inyod et al., 2022). Fruiting bodies of *M. crassa* contain Selenium (Inyod et al., 2022) which can prevent and reduce the risk of cancer (Vinceti et al., 2018). Also, this mushroom has anti-proliferative effect on the MCF7 breast cancer cell line (Pal et al., 2019). Zinc oxide nanoparticles synthesized using *M. crassa* mycelia also exhibit antimicrobial and anti-tumor activities (Suvetha and Siva, 2023). Acharya et al. (2015) stated that this mushroom contains several bioactive mycochemicals such as flavonoids, phenol, ascorbic acid, β-carotene, and lycopene, and these chemicals might be responsible for the bioactive properties of the mushroom.

*Macrocybe titans* has not been reported as a medicinal remedy in traditional pharmacopeia, but recent studies have identified some medicinal properties. Fucogalactans of *M. titans* have been identified with inhibitory activity against melanoma cell migration (da Silva Milhorini et al., 2018). In *M. titans*, a complex triglyceride is identified as responsible for anticancer properties, known as macrocybin. The mechanism of action of the macrocybin involves Caveolin-1 overexpression and actin cytoskeleton disorganization in the cancer cells (Vilariño et al., 2020). This mushroom has been identified as having antimicrobial activity against several pathogenic bacterial species, *Bacillus cereus*, *Escherichia coli*, *Klebsiella pneumoniae*, *Listeria monocytogenes*, *Pseudomonas aeruginosa*, *Salmonella enteritidis*, *Salmonella typhimurium*, and *Staphylococcus aureus* (Junior et al., 2021) and antifungal activity against the pathogen *Candida albicans* (Pereira et al., 2023). *Macrocybe titan* is also a rich source of β-D-glucan and α-D-glucan (da Silva Milhorini et al., 2022), which have different medicinal properties (Ciecierska et al., 2019; De Felice et al., 2020). β-D-Glucans have been identified as hypocholesterolaemia polysaccharides since they reduce cholesterol and bile acid concentrations in the intestinal lumen, impairing the absorption from enterocytes. This causes reduced plasma cholesterol levels (Sima et al., 2018). β-D-Glucans also can inhibit the activity of 3-hydroxy-3 methyl-glutaryl-coenzyme reductase, which is involved in the rate-limiting step of cholesterol biosynthesis (Tong et al., 2015). The glucans also show some cytotoxic effects on tumoral breast cells as well (Morales et al., 2020).

*Macrocybe gigantea* is also of several pharmacological properties. This species shows antimicrobial (Giri et al., 2012; Das, 2017; Gaur and Rao (2017); Roy et al., 2022), antioxidant (Banerjee et al., 2007; Chatterjee et al., 2011; Khatua et al., 2013; Khatua and Acharya, 2016; Das et al., 2017; Ghafoor and Niazi, 2023; Nirmala and Siva, 2023), antitumor (Dai et al., 2009), immunomodulatory (Ooi, 2001; Dai et al., 2009; Pamitha, 2014; Zhao et al., 2020; Chugh et al., 2022) and hepatoprotective (Acharya et al., 2012; Pamitha, 2014; Kumar et al., 2022) properties. Apart from that, this mushroom is rich in gallic acid Gaur and Rao (2017), an anti-HIV compound (Modi et al., 2013). Also, *M. gigantea* is rich in minerals like Cu, which is required for immunity maintenance and healthy bones, Zn, which is vital in immune maintenance, Fe, which is required for blood haemoglobin levels and Mn, which is vital in amino acid, carbohydrate, and cholesterol metabolism. Also, it is rich in K, Ca, and Mg which are beneficial for maintaining cardiovascular health conditions (Roy et al., 2022). *Macrocybe gigantea* mushrooms are rich in secondary metabolites like alkaloids, tannins, flavonoids, phenolics, and steroids, which are responsible for the bioactive properties of the mushroom species Gaur and Rao (2017); Roy et al., 2022; Nirmala and Siva, 2023). Chatterjee et al. (2014) have identified an ergosteryl triterpene, gigantenol, from this mushroom, a secondary metabolite with potential nutritional properties. An *in-silico* analysis by Roy et al. (2022), has identified the myco-compound targets of this mushroom, and the biological activities of the respective mycocompounds have been predicted. According to this study, most of the mycochemicals were responsible for anti-dihydrofolate reductase, anti-arachidonate 5-lipoxygenase LOX5, antiarrhythmic, anti-cyclin-dependent kinase CDK4, anti-COX1, and anti-HIV properties. The Ag nanoparticles produced from *M. gigantea* have also been identified with antimicrobial activity (Nirmala and Siva, 2023).

*Macrocybe lobayensis* also has been identified with antimicrobial (Giri et al., 2012), antioxidant, and immune-stimulatory (Khatua and Acharya, 2022) properties. Khatua et al. (2017) mention that this mushroom is of potential antioxidant, antimicrobial, and anti-cancer activity attributed to the presence of phenols and flavonoids. Other than these, they state that this mushroom species contains β-carotene, lycopene, ascorbic acid, p-hydroxybenzoic acid, p-coumaric acid, salicylic acid, cinnamic acid, and pyrogallol which are responsible for different medicinal properties. Furthermore, Ghosh et al. (2019) mention that hot water-soluble crude polysaccharides of this mushroom have potential antioxidative activity and immune-boosting effect on macrophage cells. According to Khatua and Acharya (2023), the hot alkali-soluble crude polysaccharide faction of this mushroom species exhibits antioxidant and strong immune-potentiation activities. The ethanolic extract of this mushroom also has antioxidant and antimicrobial potential, and the extracts are composed mainly of phenolic compounds such as pyrogallol, cinnamic acid, p-coumaric acid, and p-hydroxybenzoic acid (Khatua and Acharya, 2018).

## 9 Toxicities and related studies

Mushrooms are naturally rich in nutritional and medicinal compounds, but they can sometimes contain hazardous substances. Although *Macrocybe* species are considered edible, they often contain cyanogenic compounds, and precautions should be taken before consumption (Pegler et al., 1998). Falandysz et al. (2015) mentioned that the mushroom *M. gigantea* from Southwestern Asia contains radioactive ^137^Cs that cause different health issues, and this toxicity can be eliminated by boiling and discarding the boiled water. The main reason behind the contamination with ^137^Cs is the contamination of the soil with radioactive contaminants which enter the mushroom fruiting bodies. Although the available concentrations of these contaminants are low in mushrooms, the amount that can enter the human body in consumption can be high due to the large mushroom size. They also have identified a natural radioactive isotope, ^40^K from *M. gigantea* mushroom. Some studies have identified the presence of toxic heavy metals (Cd, Pb and Hg) in mushrooms of *Macrocybe* (Liu et al., 2012; Wiejak et al., 2014). Although *M. gigantea* is a deemed edible mushroom, consumption of this mushroom has led to several cases of gastrointestinal intoxication symptoms in China (Li et al., 2020, Li et al., 2023) and Thailand (Nooron et al., 2023). Beug (2016) states that cooked *M. spectabilis* has caused vomiting in two cases in Hawaii, although the trace amounts of cyanic compounds present in this mushroom are removed in cooking. Finnegan (2003) mentions that the mushrooms *M. titans* and *M. spectabilis* contain more cyanide than the other species of the genus and in India and other African countries, these mushrooms are boiled in water with several changes of water. Considering these, necessary precautions such as thorough cooking and washing well with water should be taken when consuming the species of this genus. These mushrooms should never be consumed raw, and thorough cooking is recommended.

## 10 Concluding remarks and future directions

*Macrocybe* comprises eight species; some are well-documented, while others are rarely mentioned in scientific literature. Misidentification is commonly observed among the species of this genus due to their closely similar morphological characteristics. Although all the mushrooms of the genus are of large mushroom size and have high palatability, cultivation has not been popularized. The main reason behind this may be the difficulty of cultivating these species. Due to the high economic potential of these mushrooms, large-scale artificial cultivation can be introduced to meet the demand. The main challenges in cultivating these mushrooms species can be summarized as the lack of knowledge on novel cultivation methods, scarcity of suitable substrate media, limited availability of spawn cultures, and difficulty in mushroom pin-head initiation. Studies should be conducted to address these challenges. Different sustainable, cost-effective substrate media and cultivation strategies can be developed and implemented to increase the yield and quality of these mushrooms. Novel biotechnological applications in mushroom cultivation, such as liquid spawns, automated inoculation, incubation and harvesting, and cultivation in controlled environments can be applied to increase the efficiency of the cultivation of *Macrocybe* species. Additionally, strain selection has not been recorded for any species in this genus. Enhancing the industrial potential of these mushrooms can be achieved by developing novel strains with improved characteristics, such as reduced toxicity, increased yield, enhanced flavor, shorter cultivation periods, and extended harvest duration. Furthermore, the main cultivation technique that is practiced by farmers to cultivate these mushroom species is the bag cultivation method. Diversifying cultivation methods to include techniques such as column cultivation, tray cultivation, mound cultivation, or log cultivation can lead to increased yields and improved quality of these mushrooms. Studies can be conducted to identify the suitability of these cultivation methods.

The nutrient content of mushrooms varies widely across different studies. Moisture content mainly depends on external moisture, which may explain the variation observed in moisture levels. The substrate on which the mushrooms grow is a key factor in determining variations in other nutrient contents. The mushroom strain may also contribute to these differences. To produce mushrooms with high nutrient quality, substrates rich in nutrients can be selected, and the substrate medium can be supplemented with various types of nutrients.

Various value-added products, such as dietary supplements and processed food items, can be produced from these mushrooms. These mushrooms have the potential to be commercialized as dried mushrooms, canned mushrooms, pickles and sausages, powdered products such as protein supplements, food seasonings, and herbal drinks. Apart from using as a food product, they also can be utilized in producing skin care products and packaging materials. Although these mushroom species are known as edibles, they contain cyanic toxins. Therefore, precautions should be taken to remove these toxins before consumption. Different processing techniques can be developed, that remove these toxins before marketing the mushroom products, and future studies could focus on identifying various mechanisms to remove the toxicity from these mushrooms. Previous studies have identified many medicinal properties of these mushroom species such as antimicrobial, antioxidant, anti-inflammatory, anticancer, and several others. Further research can explore additional medicinal properties, as well as investigate the chemical composition of the mushrooms and the compounds responsible for their medicinal properties. Attributed to these medicinal properties, there is a high potential for introducing these mushrooms to the market as nutraceuticals. Studies can be further upgraded to test these bioactive properties via higher-order animal models and through clinical trials.

Furthermore, the whole genome and transcriptome of these mushroom species have not yet been revealed. *In-silico* analyses based on genomics and transcriptomics data could be utilized to identify many additional characteristics of these mushrooms. The phylogeny of the species also can be elaborately studied based on these data and the confusion about the species complex will be able to resolve. Furthermore, specimens of *M. sardoa*, *M. pachymeres*, *M. praegrandis*, and *M. spectabilis* should be re-collected to clarify the taxonomic status of these mushrooms. Only a single specimen or a few specimens of these mushrooms have been collected and some of them only contain morphological information. Furthermore, molecular sequence data of *M. pachymeres*, *M. praegrandis*, and *M. spectabilis* are still lacking in GenBank, and therefore for a clear understanding of these species, detailed morphological and molecular characterization should be essentially conducted using new specimens to reveal the authenticity of these species. Although the genus *Macrocybe* has been well-studied, there are still gaps in our understanding related to phylogeny, distribution, nutrient content, bioactive properties, cultivation methods, and toxicities that need to be addressed to gain a more comprehensive picture of the genus.
